# The mannose receptor is expressed by subsets of APC in non-lymphoid organs

**DOI:** 10.1186/1471-2172-6-4

**Published:** 2005-02-08

**Authors:** Sheena A Linehan

**Affiliations:** 1 Sir William Dunn School of Pathology, South Parks Road, Oxford, OX1 3RE, UK; 2 Department of Infectious Diseases, Centre for Molecular Microbiology and Infection, Imperial College School of Medicine, Armstrong Rd., London, SW7 2AZ, UK

## Abstract

**Background:**

The mannose receptor (MR) is an endocytic receptor of Mφ and endothelial cell subsets whose natural ligands include both self glycoproteins and microbial glycans. It is also expressed by immature cultured dendritic cells (DC), where it mediates high efficiency uptake of glycosylated antigens, yet its role in antigen handling in vivo is unknown. Knowledge of which APC subsets express MR will assist the design of experiments to address its immunological functions. Here the expression of MR by MHC class II positive APC in non-lymphoid organs of the mouse is described.

**Results:**

MR positive APC were identified in several peripheral organs: skin, liver, cardiac and skeletal muscle and tongue. MR positive cells in salivary gland, thyroid and pancreas coexpressed MHC class II and the myeloid markers macrosialin and sialoadhesin, but not the dendritic cell markers CD11c or DEC-205. MR and MHC class II colocalised in confocal microscope images, implying that antigen capture may be the primary role of MR in these cells. Distinct ligands of MR were found in salivary gland and pancreas tissue lysates that are candidate physiological ligands of MR positive APC in these organs.

**Conclusions:**

The tissue and subcellular distribution of MR suggest it is appropriately located to serve as a high efficiency antigen uptake receptor of APC.

## Background

Dendritic cells (DC), APC specialised for the efficient stimulation of naïve T cells, are of fundamental importance in the control of antigen-specific immune responses (reviewed in [1]). Immature DC are sparsely distributed in peripheral organs, where they act as sentinels, continuously sampling the antigenic environment. They undergo maturation in response to stimuli that include microbial components and tissue damage, and migrate to T dependent areas of lymphoid organs. Here, they upregulate expression of costimulatory molecules and peptide-loaded surface MHC class I and II molecules and develop the capacity to stimulate antigen-specific T cells restricted by MHC class I and II.

Immature DC capture antigens by receptor-mediated endocytosis, in addition to macropinocytosis and phagocytosis (reviewed in [2]). DC are phenotypically and functionally heterogenous (reviewed in [3]), so the ability to target antigens via specific receptors to different subsets of DC in vivo may help to reveal distinctive features of their roles. One candidate receptor for endocytosis in DC is the mannose receptor (MR), or CD206, which recognises glycoconjugates bearing terminal mannose, fucose and N-acetylglucosamine by interaction with its carbohydrate recognition domains (CRDs). Natural ligands include microbial polysaccharides, glycoproteins and glycolipids, and mammalian glycoproteins with N-linked high-mannose (reviewed in [4]). MR is expressed mainly by subsets of Mφ and endothelial cells in vivo [5,6], but it is also expressed by immature cultured DC, where it endocytoses mannosylated ligands for processing and presentation to T cells by MHC class II [7]. Mannosylation of antigen confers a greatly enhanced efficiency of presentation to T cells of the order of 100 [8], and 200 to 10 000-fold [9]. However, MR is not expressed by immature splenic DC or epidermal Langerhans cells in situ in naïve mice [6], and its contribution to T cell immunity remains unknown.

Notwithstanding their role in stimulating immune responses, it is becoming increasingly apparent that DC play a role in maintaining T cell tolerance to self antigens in the periphery (reviewed in [10,11]). Direct evidence that DC can induce T cell unresponsiveness under non-inflammatory conditions came from an elegant study in which a model MHC class II peptide was targeted to DC in situ [12]. The peptide was engineered into a mAb specific for DEC-205 [12], an endocytic receptor of DC that is structurally related to MR [13]. Although T cells initially proliferated in vivo in response to DC targeting, the response was short-lived, and mice were rendered unresponsive to subsequent challenge with antigen in adjuvant [12]. Antigen targeting via DEC-205 also led to CD8+ T cell tolerance in the steady state [14], and the generation of regulatory T cells [15]. The latter suppressed proliferation of conventional CD4+ T cells in vitro, and appeared to exert immunosuppressive effects in both CD4+ and CD8+ T cell driven immunopathologies [15].

We recently found MR positive interstitial cells in the thyroid, pancreas and salivary gland: secretory organs which bear endogenous ligands of the CRDs of MR [16]. Thyroglobulin was identified as the major MR ligand in the thyroid. Since this glycoprotein is an autoantigen, we postulated that APC in the thyroid may use MR for antigen capture. As a basis for experiments to determine the role of MR in antigen handling in vivo, especially in secretory organs, we surveyed naïve murine non-lymphoid organs for MR positive APC and report here on their phenotypic characterisation. We also provide further evidence for the existence of distinct ligands of MR in pancreas and salivary gland. In light of the immunosuppressive function of immature DC in non-inflammatory conditions, a role for MR in antigen capture by APC for the purposes of maintenance of T cell tolerance to its ligands is credible.

## Results

We previously characterised the expression pattern of MR in the adult mouse by immunohistochemistry using a polyclonal Ab raised against MR, and by in situ hybridisation [6]. MR positive cells detected by immunohistochemistry in lymphoid organs were distinguished from the most well characterised DC subsets by their location and morphology, and by comparison with expression of DC markers in adjacent sections. Here, the MR-specific mAb MR6F3 [17] has been used in immunofluorescence and confocal microscopy to identify and characterise MR positive APC in non-lymphoid organs.

### MR expression by MHC class II positive cells in non-lymphoid organs

**Figure 1:**
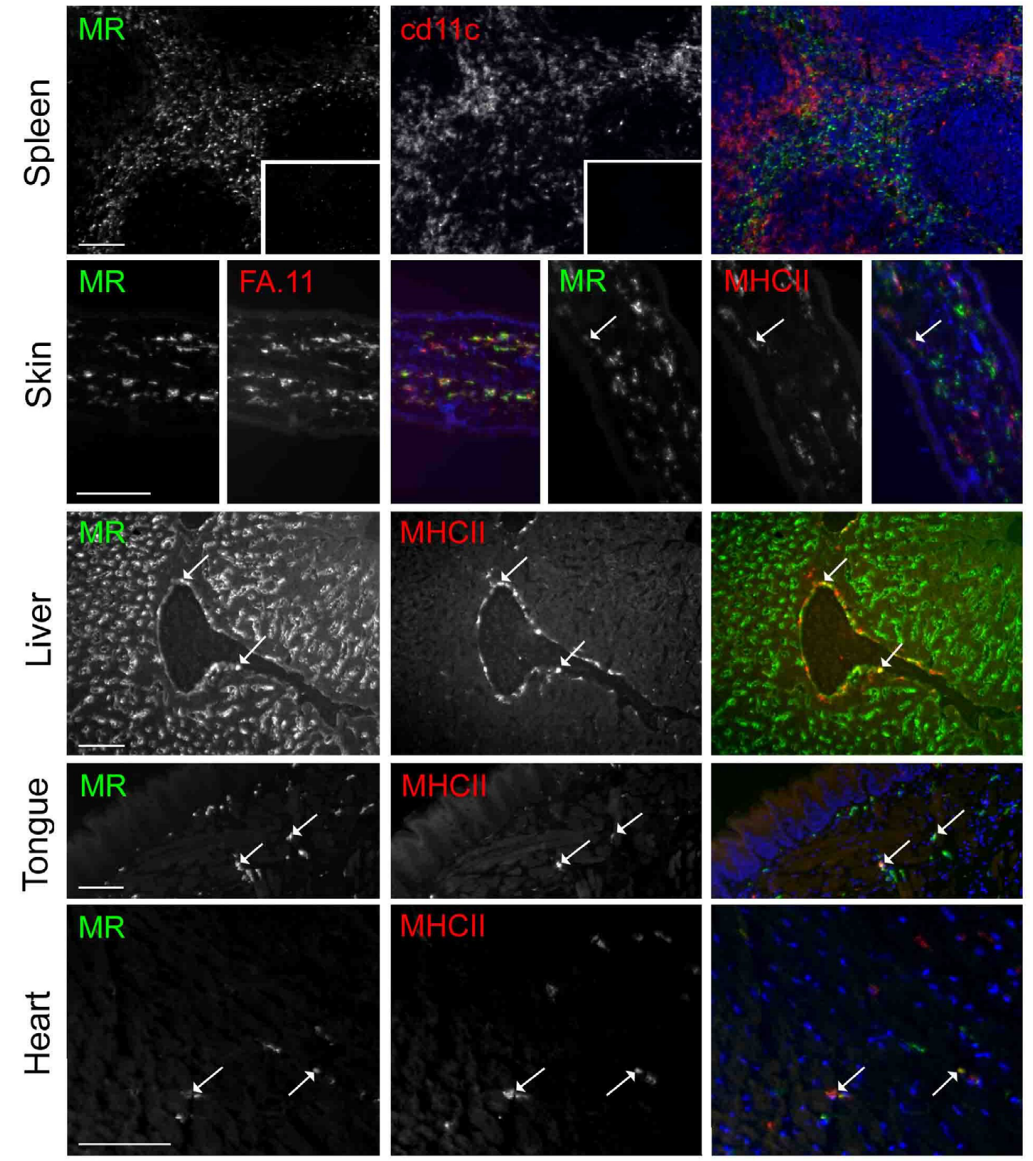
**Mannose receptor positive and negative APC subsets identified by immunofluorescence microscopy**. Tissues were examined by immunofluorescence microscopy for MR expression and other APC phenotypic markers in dual-labeled specimens as indicated. MR was detected using MR6F3_A488 _mAb. Images of each antigen labelling are shown separately in gray-scale, and merged in colour, in some cases with nuclear counterstaining shown in blue. Colocalisation of red and green is indicated by yellow. Examples of MHC class II-positive APC which coexppress MR are indicated with arrows. These constitute a subset of APC in the skin, liver, tongue and heart. No background was detected with an irrelevant IgG2a_A488 _negative control (shown in the spleen inset of MR-labelling only). Background labelling due to secondary Abs alone was also not detected in control sections (shown for anti-hamster IgG in the spleen inset of CD11c labelling only). Scale bars are indicated in MR-labelled panels only, and are 100 μm.

The specificity of MR6F3_A488_, was verified by observation of the expected expression pattern of MR in the spleen. Expression was restricted to the red pulp and did not overlap in dual-labeled specimens with the CD11c positive immature DC located at the border of the white and red pulps (figure 1). No non-specific binding of the isotype matched control antibody, IgG2a_A488 _was detected. The lack of expression of MR by DEC-205 positive DC in the thymus was also confirmed (not shown).

To detect the scattered interstitial DC of non-lymphoid organs, sections were labeled for MHC class II (figure 1). Although other APC such as Mφ can express MHC class II, constitutive expression by non-DC is absent in most organs of naïve mice. In the skin, almost complete coexpression of MR and the pan-Mφ marker macrosialin (FA.11) was found, but only a minor subset of MHC class II positive cells in the dermis coexpressed MR. DC of the hepatic portal triads are known to express MHC class II (reviewed in [18]). A proportion of such cells in the liver were found to coexpress MR. MHC class II positive cells in the tongue and heart also expressed MR, as did those in skeletal muscle (Table 1). In summary, expression of MR by MHC class II positive cells in non-lymphoid organs of naïve mice is not uncommon.

**Table 1:** Expression of Mannose receptor by DC and APC differentiated in vivo

Organ or Tissue	Cell type	Expression of MR	Species	Reference
Lymphoid organs				

Spleen	Immature and mature DC	-	Mouse	[6]
Lymph node	Interdigitating cells	-	Mouse	[6]
Lymph node	T cell area APC	+	Human	[23]
Lymph node	Follicular border APC	+	Mouse	[6]
Peyer's patch	APC	-	Mouse	[6]
Thymus	Interdigitating cells	-	Mouse	[6]

Non-lymphoid organs				

Skin	Langerhan's cells	-	Mouse, Human	[6, 22-25]
Skin (atopic dermatitis and psoriasis)	Inflammatory dendritic epidermal cells	+	Human	[25]
Skin	Dermal APC	Minor subset +	Mouse	This study
Lung	Immature DC	Endocytose MR ligand	Human	[31]
Peritoneum	DC	-	Mouse	[44]
Blood	DC	-	Human	[45]
Thyroid	Immature DC / APC	+	Pig, Mouse	[27]; This study
Pancreas	APC	+	Mouse	This study
Salivary gland	APC	+	Mouse	This study
Liver	Hepatic portal triad APC	Subset +	Mouse	This study
Muscle (cardiac and skeletal)	APC	Subset +	Mouse	This study
Tongue	APC	Subset +	Mouse	This study

### MR expression by novel APC in secretory organs

**Figure 2:**
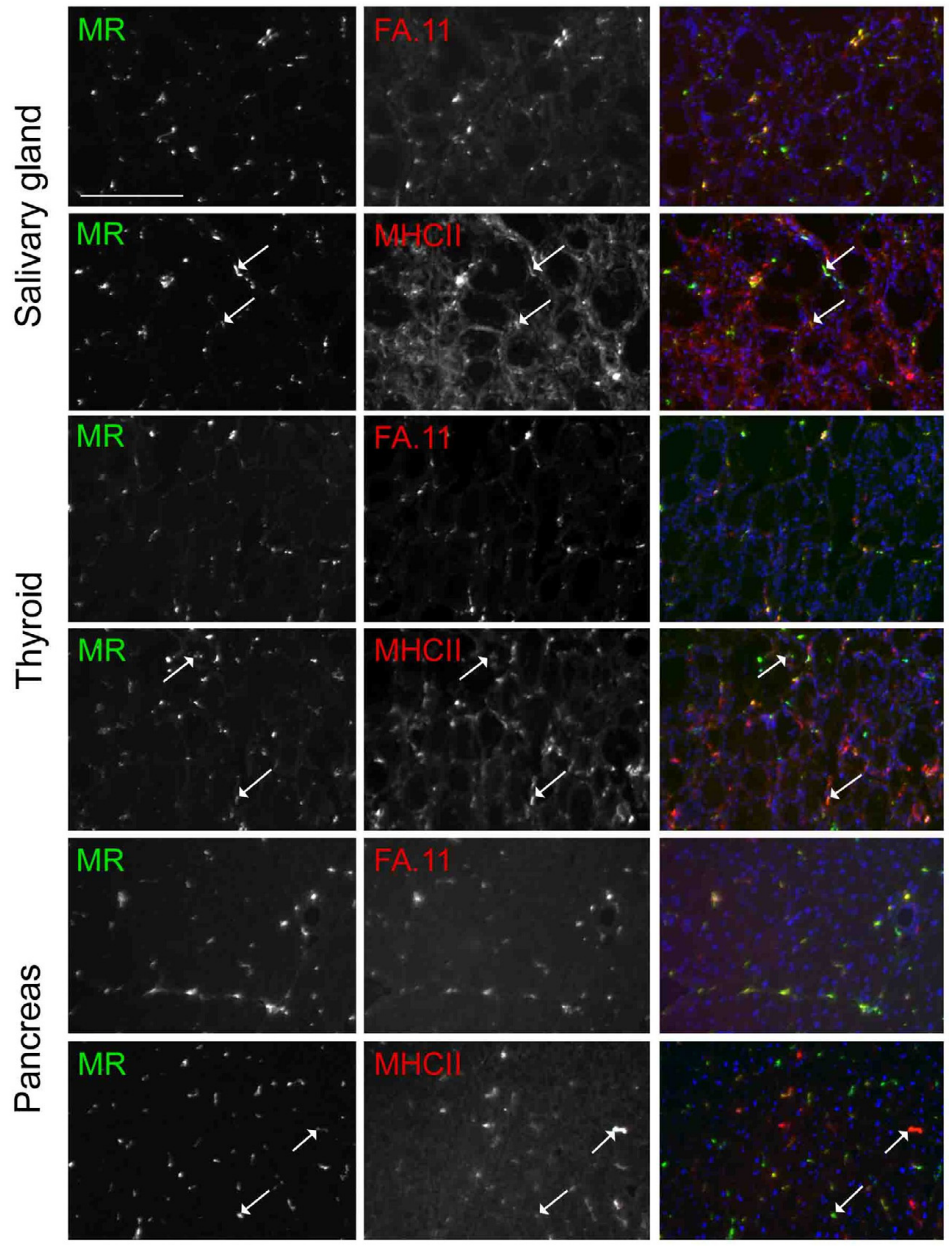
**Mannose receptor positive APC in secretory organs co-express macrosialin and MHC class II**. Secretory organ sections were probed for MR and either macrosialin (FA.11) or MHC class II as indicated. They are shown separately and as merged images that include a nuclear counterstain in blue. In merged images of MR and FA.11 labelling, there are many yellow cells, indicating colocalisation of these markers. In merged images of MR and MHC class II labelling, there are fewer yellow cells, because the fluorescence intensities of the two labels are usually very different in individual cells. However, visual comparison of singly labelled images indicates that many cells do express both markers. Examples in each tissue are indicated with arrows. The scale bar is indicated in the first panel only and is 100 μm.

We previously showed that secretory cells in the salivary gland, thyroid and exocrine pancreas were rich in ligands of the CRDs of MR, and expression of MR by adjacent interstitial cells was particularly intense [16]. In adjacent sections, we noted expression of MHC class II by interstitial cells. Here a further examination of the phenotype of MR positive interstitial cells in these organs was made. Essentially all MR positive cells coexpressed macrosialin (FA.11) (figure 2). Many if not most also expressed MHC class II, although the relative fluorescence intensities of MR and MHC class II in individual cells were often very different. In the submandibular salivary gland, MHC class II was also expressed by other interstitial cells that were probably epithelial cells. Further phenotypic analysis revealed that MR positive cells coexpressed sialoadhesin (not shown), a myeloid marker expressed by some Mφ subsets, and also some DC. Expression of DEC-205 was not detected on any MR positive cells in any of the secretory organs (not shown). Although there were CD11c positive cells in the salivary gland and thyroid, MR positive APC were negative for this marker. It was not possible to assess whether the interstitial cells of the exocrine pancreas expressed CD11c as unexpected and abundant expression of this integrin by adjacent secretory cells was found.

Since the MR positive cells of secretory organs exhibited a strongly myeloid phenotype but lacked expression of traditional DC markers, the possibility that they may be activated Mφ was considered. IFNγ is the most potent activator of Mφ, leading to expression of MHC class II, so the phenotype of interstitial cells in the secretory organs of IFNγ -/- mice was examined. Constitutive expression of MR and MHC class II was retained (not shown). In summary, MR positive interstitial cells displayed a novel APC phenotype, and constitutively expressed MHC class II in the absence of stimulation with IFNγ.

### Confocal microscopic analysis of MR positive APC in secretory organs

**Figure 3:**
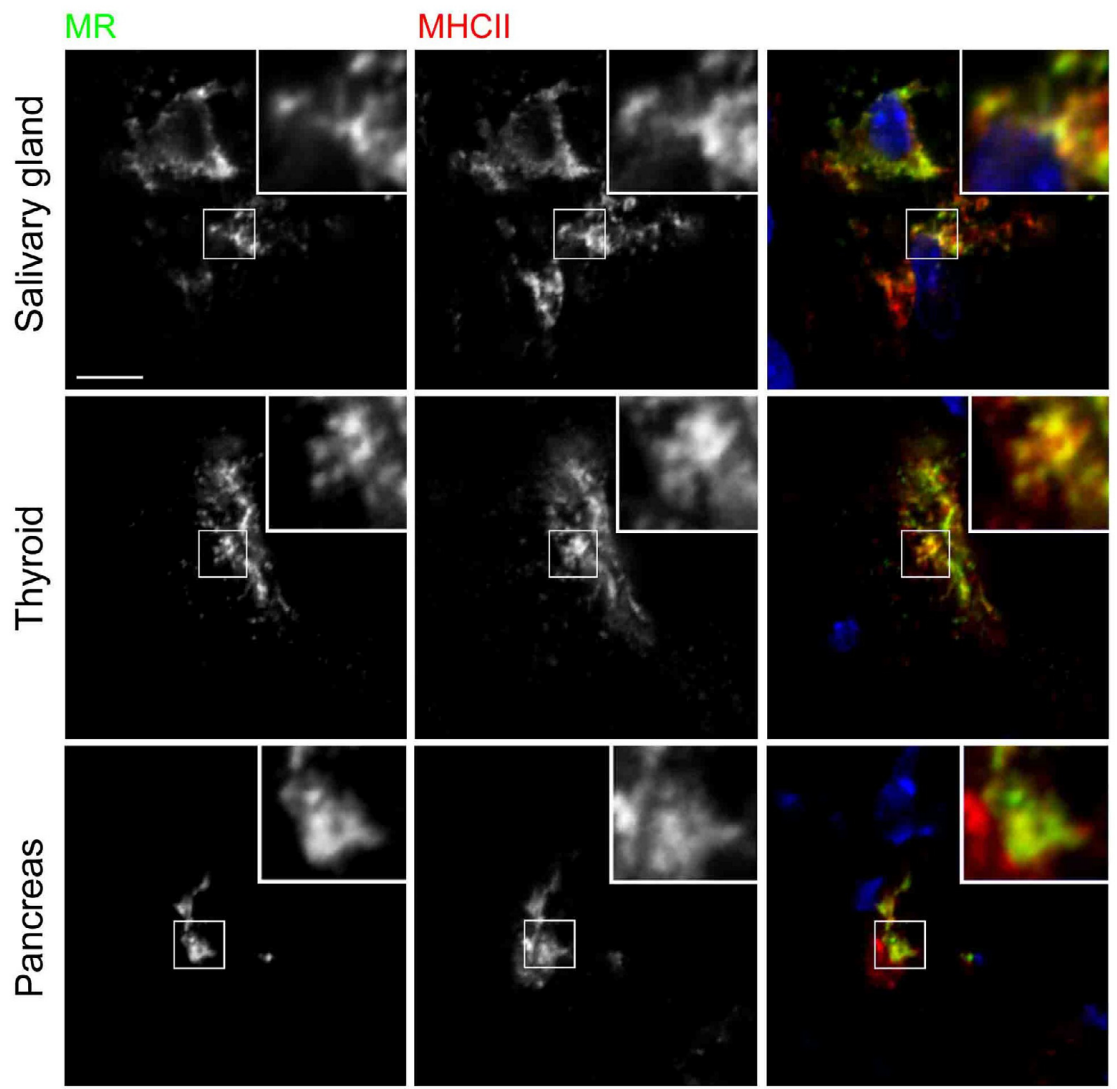
**Mannose receptor and MHC class II colocalise in confocal microscope images of secretory organ APC**. The subcellular distribution of MR and MHC class II in secretory organ APC was examined by confocal microscopy as indicated. A nuclear counterstain in blue is included in merged images. Single optical planes showing a high degree of colocalisation of MR and MHC class II were selected from z-series in which colocalisation was also observed in adjacent optical sections. Boxed regions of each image are shown at three-fold higher magnification in insets. The scale bar is indicated in the first panel only and is 5 μm.

The coexpression of MR and MHC class II in secretory organ APC implied that these cells may have immunologic functions. The distribution of these markers were examined by confocal immunofluorescence microscopy, in cells that expressed comparable levels of the two proteins (figure 3). In some cells, considerable pixel to pixel overlap of MR and MHC class II, which was not restricted to individual optical sections within z-series, was observed. The resolution of the microscope was inadequate to resolve individual vesicles, but regions of higher and lower detection of one marker were frequently paralleled by similar variations in fluorescence intensity of the other. In other cells, MR and MHC class II were not seen to colocalise, demonstrating that the resolution of the microscope and preservation of the tissues were adequate to allow different intracellular compartments to be distinguished in these cells (not shown). Furthermore, MR did not colocalise with the endosomal marker macrosialin in confocal microscope images (not shown). The results are therefore highly suggestive that MR and MHC class II are located in the same subcellular compartments within a proportion of APC.

### Characterisation of MR ligands in salivary gland and pancreas by lectin blotting

**Figure 4:**
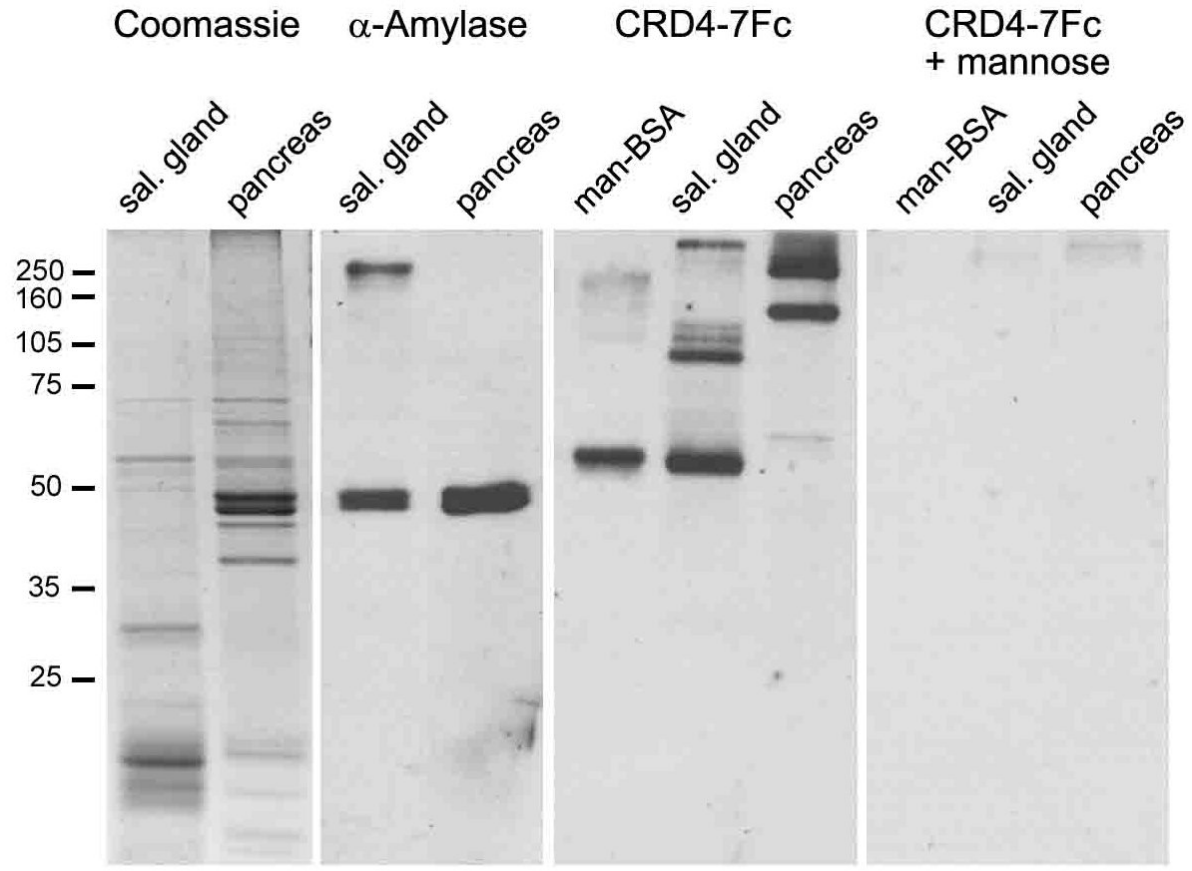
**A Mannose receptor-fusion protein recognises distinct bands in blots of salivary gland and pancreas tissue lysates**. Tissue lysates were separated by SDS-PAGE and examined by Coomassie protein staining and western and lectin blotting as indicated. The doublet of bands recognised by the antibody directed against the putative MR ligand, salivary amylase, did not correspond to the bands recognised by the MR-fusion protein, CRD4-7Fc. The specificity of this probe was confirmed by loss of all bands in blots probed with CRD4-7Fc in the presence of the competitor, mannose. Binding to mannose-BSA is shown as a positive control. Protein loaded per lane was 10 μg of salivary gland and pancreas lysates in each application except western blotting of pancreas in which 200 ng was used. 5 ng mannose-BSA was used per lane in lectin blotting. The migration pattern of molecular weight markers is indicated on the left of the image.

We previously identified thyroglobulin as an abundant ligand of MR present in the thyroid, and showed that CRD4-7Fc, an Fc-fusion protein bearing four of the CRDs of MR, which retains the same ligand specificity as MR, bound specifically to sections of salivary gland and pancreas [16]. Here, further evidence for the existence of distinct ligands of CRD4-7Fc in the salivary gland and pancreas is shown, and estimates of their Mr. Tissue lysates were separated by SDS-PAGE, transferred to nitrocellulose membranes and probed for ligands of CRD4-7Fc by lectin blotting (figure 4). Bands of approximate Mr of 55, 100, 120, 130 and >250 kD were identified from salivary gland, and bands of 60, 145, 215 and 250 kD from pancreas lysates. Binding specificity was confirmed by probing with CRD4-7Fc in the presence of mannose. Under these conditions, all binding was lost. The distribution of bands in CRD4-7Fc probed blots was compared with gels stained for protein with Coomassie. The 55 kD ligand in salivary gland may correspond to a particularly abundant fraction in the Coomassie stained gel, but other ligand bands from either organ did not appear to be associated with major protein fractions. Human salivary amylase has been shown to be a ligand of rat MR [19], so a comparison was made between the size of ligand bands and bands in blots probed for alpha amylase. Amylase was a minor component of salivary gland, although a major component of pancreas, giving rise to a doublet of bands in both with Mr of 49 and 50 kD, distinct from all bands detected in lectin blots with CRD4-7Fc. The identities of the ligands of MR in salivary gland and pancreas therefore remain unknown.

As a control, equivalent quantities of lung, thymus and spleen tissue lysates were subjected to lectin blotting with CRD4-7Fc as described above, but defined bands were not detected (not shown). We previously showed that Mφ within lung and thymus tissue sections contained ligands of CRD4-7Fc, as did Mφ and other cells within spleen [16]. The absence of defined bands in lectin blots of lysates of these tissues indicates that ligands generally present within tissue are heterogeneous in size, and that no single species is sufficiently abundant to be detectable after separation by SDS-PAGE. This is in stark contrast to the defined tissue-specific bands detected in lectin blots of salivary gland and pancreas tissue lysates.

## Discussion

The MR was the first non-opsonic C-type lectin receptor shown to be expressed and mediate adsorptive endocytosis in DC [7,20], properties it shares with a growing family of such receptors (reviewed in [21]). Expression of MR by DC appears to be tightly regulated. In human peripheral blood monocyte-derived DC, MR expression is down-regulated by inflammatory stimuli, and for this reason it has been considered a prototype marker of immature DC [7]. However, human epidermal Langerhans cells which are functionally immature, do not express MR in situ [22-25], although have been found to be positive for MR after a short isolation procedure [26]. In fact, many DC subsets that have differentiated in vivo do not express MR; a summary of MR expression by APC is shown in table 1. Here, the expression of MR by APC in non-lymphoid organs of the naive mouse is described. Cells were analysed in situ, circumventing the problem that isolation or culture of cells for ex vivo analysis may affect expression of MR.

In contrast to our earlier observation that MR is not expressed by well-characterised subsets of DC in lymphoid organs [6], many APC in peripheral tissues expressed MR, including a proportion of MHC class II positive cells in skin, liver, tongue and heart. Whilst a limited phenotypic analysis is not sufficient to prove that these cells are DC, in the case of MR positive cells in the thyroid, pancreas and salivary glands, this designation appears to be a strong possibility. DC are normally distinguished from monocytes and macrophages by phenotype, morphology and function, properties which are most amenable to study after cell isolation. MR is expressed by interstitial cells in situ in pig thyroids, and MR positive cells have been successfully isolated from this source, and maintained in co-culture with thyrocytes in the presence of thyroid stimulating hormone [27]. Isolated cells expressed MHC class II, S100 protein and had a high endocytic capacity, characteristic of DC. They also exhibited morphological features of DC including a plasma membrane with numerous processes, a reniform nucleus and intracellular annular structures. In response to a stimulator of DC maturation, TNFα, the cells detached from the culture substratum and lost the ability to endocytose the MR ligand, FITC-dextran. By extension, the MR positive APC detected in the murine thyroid may also be immature DC. MR positive APC in the pancreas and salivary gland were phenotypically indistinguishable from the thyroid cells, according to the markers used in this study. APC in all three secretory organs exhibited a strongly myeloid phenotype, expressing macrosialin and sialoadhesin, but were unlikely to be activated Mφ since they were also present in IFNγ -/- mice. MR positive immature DC or other APC at peripheral sites are likely to down-regulate MR when they mature and migrate to lymph nodes, in accordance with in vitro models [7,27], since the mature interdigitating cells of tissue-draining lymph nodes do not express MR [6].

The function of MR in relation to antigen handling in vivo is poorly understood, in part because most studies have used glycosylated Ag that are now known to be ligands of other receptors in addition to MR (reviewed in [28]). However, a specific role for MR in enhancing uptake and presentation to both CD4^+ ^and CD8^+ ^T cells by DC has recently been shown using Ag fused to mAb specific for MR [29,30]. Surprisingly, reports of any ability of MR to enhance primary immune responses in vivo have not been forthcoming, but since MR is not expressed by splenic DC or epidermal Langerhans cells (figure 1; [6]), MR may not play a role in DC capture of antigen administered intravenously or by skin absorption. Isolated human lung DC exhibit a high endocytic capacity for the MR ligand, FITC-dextran [31]. Therefore, MR may play a role in handling of inhaled antigens, such as the house dust mite allergen, Der p 1, whose endocytosis by DC is dependent on MR [32]. A link between MR and allergy has been suggested based on the finding that monocyte-derived DC from allergic patients expressed more MR and endocytosed Der p 1 more efficiently than did DC derived from healthy donors [32].

Antigen uptake by MR on cultured DC promotes presentation to T cells of antigen-derived peptides on MHC class II [7]. The colocalisation of MR and MHC class II in confocal microscope images of APC in thyroid, pancreas and salivary gland described here implies a role in antigen uptake for presentation. Interestingly, in mouse bone marrow-derived DC and a MHC class II-expressing fibroblast cell line, MR did not colocalise with MHC class II [33]. However, the structurally similar receptor, DEC-205, did target MHC class II compartments, and this was found to depend on the presence of an EDE triad within the receptor's cytoplasmic tail, which MR lacks. When fused to the extracellular domain of the Fc receptor, the cytoplasmic tail of DEC-205 directed endocytosed human IgG to a compartment from which MHC class II loading and subsequent presentation of IgG-derived peptides was 100-fold more efficient than that driven by the cytoplasmic tail of MR. In another study, efficiencies of processing and presentation of ribonucleases A and B by a fibroblast cell line expressing MR and MHC class II IA^K ^were compared [34]. These model Ag differ only by the presence of a high-mannose oligosaccharide on RNAse B. Although RNAse B, a ligand of MR, was endocytosed more efficiently than the non-ligand RNAse A, efficiencies of presentation of the two Ag were indistinguishable [34]. Therefore, MR does not constitutively enhance Ag presentation of its ligands. To do so, it may need to target MHC class II compartments. The apparent colocalisation of MR and MHC class II in secretory organ APC implies that the intracellular locations of MR or MHC class II molecules in these cells may be distinct from those in bone marrow-derived DC and transfected fibroblasts, perhaps permitting MR to target Ag to these compartments with greater efficiency. Unusually for immature DC, those cultured from pig thyroid were characterised by plasma membrane expression of MHC class II [35].

If MR is poised to direct Ag to the MHC class II compartments of secretory organ APC, the question of which Ag are captured in these environments must be raised. We previously showed that thyroglobulin, a major auto-antigen in murine autoimmune thyroid diseases, is a ligand of MR, and suggested that MR expressed by interstitial cells in the thyroid may be involved in maintaining tolerance to this Ag in normal mice [16]. Here, further evidence is presented that the pancreas and salivary glands also bear abundant MR ligands. A high molecular weight fraction in salivary gland lysate (>250 kD) may correspond to secretory IgA. This Ab inhibits adhesion of microbes to the gut wall, but is a non-inflammatory isotype, to avoid unwanted immune reactions against indigenous microflora and dietary Ags (reviewed in [36]). Uptake of secretory IgA by cultured human DC could be partially blocked by mAb against MR and this pathway has been suggested to allow modulation of mucosal immune responses [37]. The MR ligands in pancreas lysate were of different Mr to those in salivary gland, and remain undefined. A role for MR in maintenance of immune tolerance to its ligands in this organ is credible since pancreatic DC of naïve mice are believed to be tolerogenic [38]. DC isolated from the lymph node draining the pancreas, but not from other lymph nodes, were able to confer protection against diabetes when transferred to the nonobese diabetic (NOD) mouse strain [38].

In light of the location of MR positive APC in secretory organs, the abundance of endogenous ligands, and the positive identification of one ligand as thyroglobulin, the available data could suggest a tolerogenic role of MR is more likely than an immunostimulatory role in these organs in the steady-state. Such a mechanism may represent a physiological correlate of the experimentally induced immune tolerance achieved by targeting Ag to DEC-205 in naïve mice [12,14,15]. Further studies will be required to address the significance of MR to immune homeostasis.

## Conclusions

MR positive APC are present in non-lymphoid organs of the naïve mouse, but in most organs examined, only a subset of MHC class II positive cells express MR, in contrast to the ubiquitous expression of MR described in cultured DC. This information will inform the design of experiments to test the function of MR in antigen handling in vivo. An immuno-regulatory role for MR in relation to its endogenous ligands in salivary gland, pancreas and thyroid is suggested by the finding that MR positive APC are abundant in these organs, they co-express MHC class II and appear to have an overlapping subcellular distribution of MR and MHC class II.

## Methods

### Mice

IFNγ -/- mice [39] were backcrossed for 14 generations onto the C57BL/6 background at the Sir William Dunn School of Pathology, Oxford, UK. IFNγ -/- and WT C57BL/6 mice were bred and used at 8 to 12 weeks of age in accordance with Home Office legislation. No differences in phenotype or abundance of MR positive APC were noted between male and female mice.

### Antibodies and Fc-fusion protein

Rat mAb were prepared in our laboratory and used at optimal concentrations for immunolabelling. They were directed against MHC class II (clone TIB120; ATCC); macrosialin (clone FA.11; [40]) and sialoadhesin (clone 3D6; [41]). Rat mAb against DEC-205 (clone NLDC-145; [42]) and hamster mAb against CD11c (clone N418; [43]) were purchased from Serotec (Kidlington, UK). MR6F3 directed against MR [17], and rat IgG2a were conjugated to Alexa_488 _(Molecular Probes, Leiden, The Netherlands) according to the manufacturer's instructions. Goat anti-amylase was from Santa Cruz Biotechnology Inc (Santa Cruz, CA). CRD4-7Fc was prepared as described [16]. Secondary Abs were goat F(ab')_2 _anti-rat IgG, and goat F(ab')_2 _anti-Armenian hamster IgG, both cy3 conjugated, and horseradish peroxidase conjugated mouse F(ab')_2 _anti-human IgG Fc (Jackson ImmunoResearch Labs; West Grove, PA). Horseradish peroxidase conjugated donkey F(ab')_2 _anti-goat IgG was from Chemicon (Harrow, UK).

### Immunofluorescence labelling and microscopy

For immunofluorescence microscopy, 6 μm cryosections were prepared from unfixed frozen tissues. They were permeabilised in an incubation buffer consisting of 0.5% BSA and 0.05% saponin in PBS, then blocked with 5% normal rabbit serum in incubation buffer. Sections were subsequently incubated with rat or hamster mAb specific for APC antigens for 1 hr, then the appropriate cy3-conjugated secondary Ab for 30 min. Sections were blocked with 100 μg/ml rat IgG for 30 min, before being probed with Alexa_488 _conjugated MR6F3 mAb or isotype matched control. Nuclei were labeled with Hoechst 33342 dye (Molecular Probes). Sections were mounted in Aqua Polymount (Polysciences, Inc., Warrington, PA). Slides were examined by fluorescence microscopy and 12-bit digital images captured using a CCD camera attached to a Zeiss Axioplan photomicroscope. Slides were prepared in the same way for confocal microscopy, except that cryosections were cut at 20 μm. Confocal microscope z-stack images were collected using a Bio-Rad Radiance 2000 MP laser scanning confocal microscope, with lasers exciting at 367, 488 and 543 nm. Images corresponding to each fluorophore were collected using individual lasers sequentially to eliminate bleed-through. Immunofluorescence and confocal images were processed using MetaMorph version 4.5 software.

### Western and lectin blotting

Mouse tissue lysates were prepared in lysis buffer (2% v/v Triton X-100, 10 mM Tris pH 8.0, 10 mM NaN_3_, 150 mM NaCl, 10 mM EDTA, 5 mM iodoacetamide, 1 mM PMSF, 1 mg/ml pepstatin, 1μM leupeptin). Proteins were quantified in duplicate using Bicinchoninic acid protein assay kit (Pierce Chemical Company, Chester, UK). Samples were electrophoresed by SDS-PAGE, transferred to nitrocellulose membranes and subjected to western or lectin blotting using standard methods [16]. CRD4-7Fc was used at 1 μg/ml, with or without 100 mM D-mannose as a specificity control. Membranes were probed with the appropriate horseradish peroxidase-conjugated secondary Ab, and signal was developed using enhanced chemiluminescence (Amersham Life Science Ltd., Bucks., UK). A calcium containing buffer was used in lectin blotting throughout (150 mM NaCl, 10 mM Tris pH 7.4, 10 mM CaCl_2_) and mannose-BSA (Sigma, St. Louis, MO) was used as positive control. Gels were also stained with Coomassie R-250 to detect protein.

## Abbreviations

CRD, carbohydrate recognition domain; DC, dendritic cell; MR, mannose receptor
